# Bubble Printing of Liquid Metal Colloidal Particles for Conductive Patterns

**DOI:** 10.3390/nano14201665

**Published:** 2024-10-17

**Authors:** Masaru Mukai, Tatsuya Kobayashi, Mitsuki Sato, Juri Asada, Kazuhide Ueno, Taichi Furukawa, Shoji Maruo

**Affiliations:** 1 Faculty of Engineering, Yokohama National University, 79-5 Tokiwadai, Hodogaya-ku, Yokohama 240-8501, Japan; mukai-masaru-vw@ynu.ac.jp (M.M.); ueno-kazuhide-rc@ynu.ac.jp (K.U.);; 2 Graduate School of Engineering Science, Yokohama National University, 79-5 Tokiwadai, Hodogaya-ku, Yokohama 240-8501, Japan

**Keywords:** bubble printing, femtosecond laser, laser direct writing, liquid metal, eutectic gallium–indium alloys, conductive patterns, flexible wires

## Abstract

Bubble printing is a patterning method in which particles are accumulated by the convection of bubbles generated by laser focusing. It is attracting attention as a method that enables the high-speed, high-precision patterning of various micro/nanoparticles. Although the bubble printing method is used for metallic particles and organic particles, most reports have focused on the patterning of solid particles and not on the patterning of liquid particles. In this study, liquid metal wiring patterns were fabricated using a bubble printing method in which eutectic gallium‒indium alloy (EGaIn) colloidal particles (≈diameter 0.7 µm) were fixed on a glass substrate by generating microbubbles through heat generation by focusing a femtosecond laser beam on the EGaIn colloidal particles. The wiring was then made conductive by replacing gallium oxide, which served as a resistance layer on the surface of the EGaIn colloidal particles, with silver via galvanic replacement. Fine continuous lines of liquid metal colloids with a line width of 3.4 µm were drawn by reducing the laser power. Liquid metal wiring with a conductivity of ≈1.5 × 10^5^ S/m was formed on a glass substrate. It was confirmed that the conductivity remained consistent even when the glass substrate was bent to a curvature of 0.02 m^−1^.

## 1. Introduction

Laser processing technology has many excellent features, such as non-contact processing, high-precision processing on the micrometer to sub-micrometer scale, and high-speed scalable processing [1,2,3,4], and is used in a variety of fields, including electronics [5,6], medical applications [7,8], three-dimensional (3D) printing [9,10,11], and nanomaterial synthesis [12]. Laser processing technologies can be classified into several types, such as those that utilize photochemical reactions, heat generated by light, and light pressure [13]. Typical methods based on photochemical reactions, stereolithography, and two-photon lithography, which solidify liquid resin or a slurry mixed with resin and nanoparticles through a photopolymerization reaction, are used to create 3D structures on the micro- to macroscale using various types of lasers, such as ultraviolet lasers, blue lasers, and femtosecond pulse lasers [9,14,15]. As a method using light-induced heat, selective laser sintering, a 3D printing method based on powder bed fusion, is used to melt powder materials with heat and then solidify them to create a variety of 3D structures made of resins, metals, ceramics, etc. [10,11,15]. Therefore, laser-based 3D printing technologies such as stereolithography and laser sintering are used in a wide range of fields, including medicine, dentistry, industry, photonics, and micro-electro-mechanical systems [9,16,17].

Optical manipulation can manipulate and assemble micro-/nanoparticles using optical radiation pressure, without photochemical reactions or heating by light [18,19]. Although optical manipulation can manipulate single micro and nanoparticles, it is not suitable for the versatile or high-speed patterning of various particles, because the magnitude and direction of the optical radiation pressure depend on the material properties and size of the particles [18]. Therefore, microbubble-assisted printing has attracted attention as an alternative method for accumulating micro-/nanoparticles using a laser beam [13]. This method, also called bubble printing [20,21], bubble-pen lithography [22], and laser-induced microbubble technology [23,24], utilizes a phenomenon in which a laser beam is focused in a liquid to generate bubbles, and the flow formed around the bubbles causes the particles to aggregate [13]. A wiring speed of 10 mm/s has also been demonstrated [20], and the patterning of various particles such as metals [19,21,23], polymers [20,22,24], and quantum dots has been reported [20,22]. The high-precision patterning of submicron particles at a single-particle resolution has also been achieved [22]. However, previous studies have only demonstrated the patterning of solid particles, and the arrangement of liquid colloidal particles has not been demonstrated.

In this study, liquid metal colloids were patterned onto a glass substrate using microbubble-assisted printing to form electrically conductive lines. In many conventional microbubble-assisted printing methods, bubbles were generated by locally heating a substrate on which a thin metal film was formed by absorbing a laser beam [13,20,21,22,23]. However, because the metal-coated substrate was conductive, even if a pattern of conductive particles was fabricated on it, the wiring could not be directly formed. Several studies on patterning by bubble printing on non-conductive substrates have been reported [23,24,25,26,27]. For example, Nishiyama et al. demonstrated a method to generate microbubbles by utilizing the absorption of metal nanoparticles generated by an in situ photoreduction with a focused laser beam, and deposited various particles such as SiO_2_, TiO_2_ and Fe_2_O_3_ on a CaF_2_ substrate [26]. Edri et al. (2021) demonstrated bubble printing by irradiating a laser beam along platinum wiring formed on a glass substrate by photoreduction, depositing SiO_2_ particles on the metal wiring to form a multilayer structure [27]. In addition, bubble printing using particle absorption has been used to form fine conductive patterns [23,24]. For example, Armon et al. demonstrated conductive line patterns of Ag nanoparticles with a minimum line width of 1.7 µm [23]. Edri et al. (2020) also formed submicron wires with a minimum line width of 650 nm with polyaniline particles, a type of conductive polymer [24]. Table S1 compares this study to previous studies on bubble printing using the aforementioned conductive materials.

In our method, we also utilize the absorption of liquid metal colloidal particles to generate microbubbles and form conductive patterns on a non-conductive glass substrate. In our experiments, a colloidal dispersion consisting of eutectic gallium–indium alloys (EGaIn, 75% Ga, and 25% by weight), which were expected to have high electrical conductivity (3.4 × 10^6^ S/m [28]), was used. EGaIn has a melting point of 16 ^°^C, is liquid at room temperature [29] and has high biocompatibility [30]. Therefore, a wiring pattern consisting of EGaIn colloidal particles was suitable for the flexible wiring required in wearable sensors and biomedical devices. However, liquid metal colloids printed using microbubble-assisted printing were insulated from each other because of the oxide films on their surfaces. Therefore, the oxide film on the liquid metal colloid was removed by applying an external mechanical force [31], laser sintering [32], and acoustic field to make it conductive [33]. In this study, EGaIn colloidal particles patterned by bubble printing were immersed in a silver nitrate solution and the oxide film on the surface of the EGaIn particles was replaced with silver via galvanic replacement to produce conductive wiring. This method was simpler than conventional activation methods, as it only required immersion in liquid and had the advantage that the entire colloid surface could be activated uniformly. Furthermore, compared to methods for forming flexible liquid-metal wiring, including direct writing [34], screen printing [35], and vacuum filling in microchannels [36], this method had the advantage that wiring patterns of various line widths and shapes can be easily formed by controlling the laser intensity and scanning pattern, making it useful as an automatic method for forming complex wiring patterns.

In this study, we first evaluated the particle size of the prepared EGaIn colloidal particles using dynamic light scattering (DLS) and a scanning electron microscope (SEM) and confirmed that the particles were less than 1 µm in size. Additionally, energy-dispersive X-ray (EDX) analysis confirmed that the colloidal particles consisted of Ga and In. Next, bubble printing with the EGaIn colloidal particles was performed using a custom-built printing system equipped with a femtosecond laser. The EGaIn colloidal particles aggregated along the laser scan trajectory. EDX measurements indicated that the aggregated patterns contained Ga, confirming that liquid metal could be drawn by bubble printing and that arbitrary shapes could be patterned by laser scanning. Because the EGaIn colloids possess an insulating oxide film on their surfaces, the patterned structures are non-conductive and are unsuitable for wiring. Therefore, the oxide film was converted into a conductive metal film through galvanic replacement to render the structures conductive. We investigated the optimal galvanic replacement processing time and silver nitrate solution concentration for wiring applications and determined the conditions that achieved both electrical conductivity and flexibility. In the experiments, the conductivity was calculated using the resistance measured from the four-terminal measurements along with the cross-sectional area and length of the formed patterns. Additionally, flexibility was assessed based on the curvature and resistance values of the bent glass substrates. The line widths of the formed patterns were examined by varying the laser power.

## 2. Materials and Methods

### 2.1. Materials

*N*-methyl pyrrolidone (NMP) and silver nitrate (AgNO_3_) were purchased from FUJIFILM Wako Pure Chemical Co. (Osaka, Japan). Gallium–indium eutectic was purchased from Sigma-Aldrich Co., LLC (St. Louis, MO, USA). Gold wires (diameter 0.1 mm) were obtained from Nilaco Co. (Tokyo, Japan). Conductive adhesive (Dotite D-500) was purchased from Fujikura Kasei Co., Ltd. (Tokyo, Japan). A surface-mounted device light-emitting diode (SMD LED, EIL33-3L) was obtained from OptoSupply Limited (Hong Kong, China). Flexible glass with the trade name G-leaf (thickness: 100 µm) was obtained from Nippon Electric Glass Co., Ltd. (Shiga, Japan).

### 2.2. Characterization

The EGaIn colloidal particles in the dispersion were analyzed using dynamic light scattering (DLS, ELS-Z2, Otsuka Electronics Co., Ltd., Hirakata, Osaka, Japan). The scanning electron microscope (SEM) images were obtained using a JSM-6060LV microscope (JEOL Ltd., Akishima, Tokyo, Japan). Energy-dispersive X-ray (EDX) spectra and mapping images were obtained using SU8010 (Hitachi High-Tech Corp., Minato-ku, Tokyo, Japan). The SEM observation and EDX analysis of the samples were gold deposited. Particle size measurements using SEM images were performed using ImageJ software (version 1.54, NIH, Bethesda, MD, USA). The conductivity of the fabricated lines was calculated using the resistance and size of the object. A gold wire was attached to the fabricated line using conductive adhesive. The observation and size (length and width) of the fabricated line were evaluated using a VHX6000 microscope (Keyence Corp., Osaka, Japan). The height of the fabricated line was measured using a laser scanning microscope (VK-X250; Keyence Corp., Osaka, Japan). The resistance of the object was then measured using a digital multimeter (Agilent 34410A 6 1/2 digital multimeter, Agilent Technologies, Inc., Santa Clara, CA, USA) in a four-terminal sensing mode.

### 2.3. Bubble Printing System

**Figure 1:**
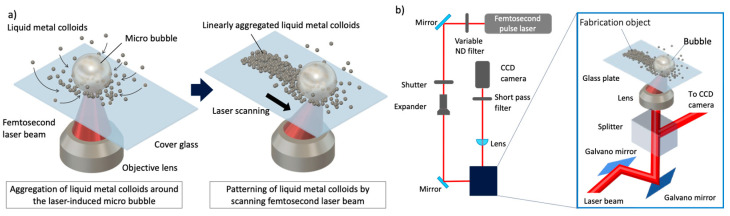
Working principle and optical system of bubble printing. (**a**) Working principle, (**b**) Schematic of the bubble printing system.

Figure 1a shows the working principle of the bubble printing of liquid metal colloids. In this method, a femtosecond laser beam focused onto a glass substrate is absorbed by liquid metal colloids on the substrate, generating localized heat. The local heating generates microbubbles, and liquid metal colloids are attracted around the microbubbles by thermal and Marangoni convection. When a focused spot is scanned on the glass substrate, the microbubbles follow the focus, and a pattern of aggregated liquid metal colloids is formed along the laser scanning path.

To demonstrate bubble printing, we constructed an optical system using a femtosecond laser, Galvano scanners, and an XYZ stage (Figure 1b). In our laboratory, we have developed several types of stereolithography systems, such as a blue laser scanning stereolithography system [37,38,39,40] and two-photon lithography systems with a femtosecond laser [41,42]. In this study, a bubble printing system was developed using basic two-photon lithography technology. The detailed setup of this system is as follows. Laser light emitted by a femtosecond laser (Mai Tai VF, Spectra-Physics Inc., Milpitas, CA, USA) with a wavelength of 752 nm was adjusted using a variable neutral density (ND) filter. The laser was turned on and off with an automatic open/closed shutter. The laser diameter was then expanded using a beam expander (magnification: 10×) and laser scanning was performed using a galvanometer mirror (GM-1015, Canon Inc., Tokyo, Japan). The galvano mirror reflected laser beams according to 2D patterns. Next, after passing through the splitter cube, the laser beam was focused by an objective lens with a numerical aperture of 0.65 onto the glass substrate on which the EGaIn colloidal particle solution was dropped. In addition, the laser beam reflected from the substrate was reflected by the splitter cube, passed through the short pass filter, and focused on a charge-coupled device (CCD) camera. The laser scanning began after the focal position was adjusted to the upper surface of the glass substrate using an XYZ stage (OSMS20-85(XYZ), SIGMAKOKI Co., Ltd., Hidaka, Saitama, Japan). To form large-scale patterns that exceed the field of view of the objective lens, after scanning with the galvano mirrors, the XYZ stage is driven and the next area is drawn. After completing bubble printing, a washing process was performed to remove excess EGaIn colloidal particles. Thus, only the particles aggregated by the bubbles generated through laser irradiation adhered to the substrate and remained, allowing for the formation of any pattern during the laser scan.

### 2.4. Preparation of EGaIn Colloidal Particles

The EGaIn colloidal particles were prepared according to the method described by David et al. [43]. NMP (15 mL) and EGaIn (1 g) were placed in a 20 mL vial. The sample was then fixed to a probe-type ultrasonic irradiation device (SONIFIER 250, BRANSON Co., Danbury, CT, USA) and irradiated at a duty cycle of 1 s and output of 3 for 20 min at 0 °C.

## 3. Results and Discussion

### 3.1. Characterization of EGaIn Colloidal Particles

**Figure 2:**
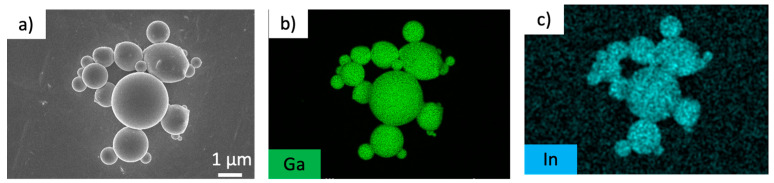
EDX mapping analysis of EGaIn colloidal particles. (**a**) SEM image, (**b**) gallium mapping image, and (**c**) indium mapping image.

Dispersions of EGaIn colloidal particles in NMP as the solvent were used. EGaIn colloidal particles were obtained by sonicating bulk EGaIn mixed in a solvent, and dynamic light scattering (DLS) measurements confirmed that the particles had an average diameter of approximately 0.7 ± 0.2 µm (Figure S1). On the other hand, the average size of the prepared particles measured by SEM was approximately 0.8 ± 0.3 µm (Figure S1 and Figure 2a). The particle size obtained from the SEM measurements was slightly larger than that obtained using DLS, possibly owing to the fusion or flattening of the particles when placed on the SEM substrate. David et al. used a similar method to prepare EGaIn colloidal particles and obtained good agreement with an average particle size of 1 µm [43]. Furthermore, EDX mapping of the particles obtained by SEM confirmed that they were composed of gallium (Figure 2b) and indium (Figure 2c). The slightly blurred contrast of the indium mapping compared to that of the gallium mapping is probably because of the high proportion of gallium in EGaIn. It is suggested that the particles consisted of EGaIn. This result indicates that a dispersion consisting of EGaIn colloidal particles was obtained.

### 3.2. Bubble Printing of EGaIn Colloidal Particles

The dispersion of EGaIn colloidal particles on a cover glass was irradiated by a 752 nm femtosecond laser beam. The galvano mirrors for laser scanning and XYZ stage were controlled by a computer, and the experiment was recorded using a CCD camera. The laser beam was focused on the interface between the substrate and dispersion solution with a lens to form a focal point. Microbubble generation was observed in the EGaIn colloidal particle dispersion (Supplemental Video S1), while no bubbles were observed in NMP alone (Supplemental Video S2). This is considered to be caused by the absorption of the laser beam by the EGaIn colloidal particles, which generates heat and microbubbles. Bubbles were observed even when water was used as a dispersant instead of NMP; however, large amounts of bubbles were generated and stable modeling was difficult (Supplemental Video S3). Nishiyama et al. used a water–ethanol mixture as a solvent for bubble printing and concluded that high fabrication accuracy could not be obtained with water alone and that the addition of ethanol, which has low evaporation energy, facilitates bubble generation and high fabrication accuracy [26]. On the other hand, Edri et al. stated that the use of NMP, which is a solvent with a high boiling point (202 °C), makes it easy to control microbubbles [24,27]. Both Nishiyama et al. and Edrin et al. appear to reach conflicting conclusions, although they argue about the ease of vaporization. NMP was more appropriate than water for this study, but it would be difficult to discuss solvents for bubble printing based simply on the ease of evaporation alone. Bubble generation is related to surface tension. As water is a liquid with high surface tension (72.14 mNs/m^2^ at 25 °C [44]), the generation of bubbles with small diameters requires greater internal pressure owing to Laplace pressure.

**Figure 3:**
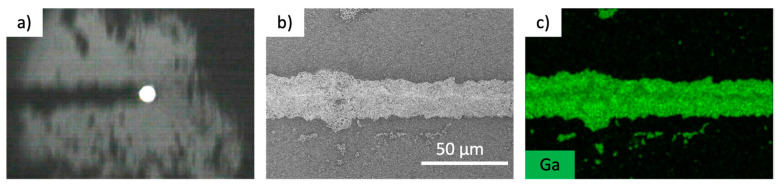
Picture of (**a**) fabrication process in progress using the EGaIn colloidal particles, (**b**) SEM image and (**b**) EDX gallium mapping image of fabricated line and (**c**) EDX gallium mapping image of fabricated line.

**Figure 4:**
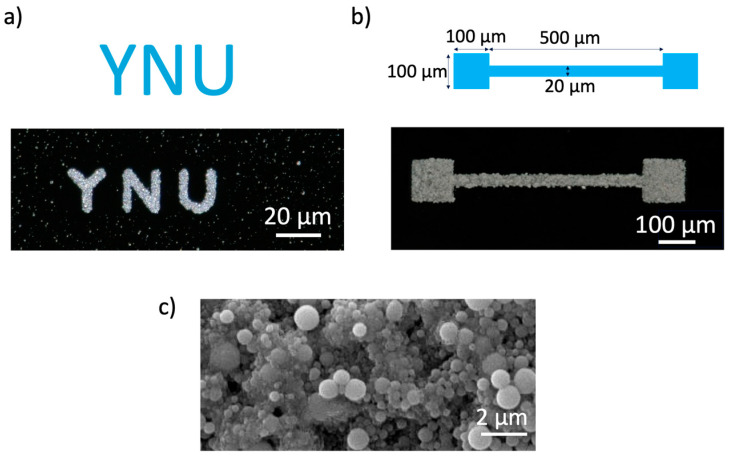
Examples of 2D pattern fabrication using EGaIn colloidal particles: (**a**) YNU, (**b**) dumbbell pattern and (**c**) high magnification SEM image of aggregated EGaIn colloidal particles.

Figure 3a shows the results of scanning the laser from left to right. Agglomerated particles were observed after the laser scanning, while bubbles were generated in the center of the image (Supplemental Video S1). It is inferred that the EGaIn colloidal particles are fixed at the interface between the bubble and substrate owing to the convection of the solvent around the bubble (Figure 3b,c). To form the pattern, the focus on the substrate was moved (800 µm/s) along a predetermined path using a galvanometer mirror to form the line pattern. Figure 4a shows a camera image of a 20 µm wide model formed using a laser power of 50 mW. An EDX mapping of the agglomerated structure of the particles confirmed that it was derived from gallium and indium, and thus, it was confirmed that it was an agglomerated structure of EGaIn colloidal particles. The laser scanning microscopy results show that the fabricated line is approximately 1 µm thick (Figure S2), and approximately the same as the diameter of the EGaIn colloidal particles.

Next, YNU (Figure 4a) and dumbbell (Figure 4b) patterns were fabricated to confirm that patterns could be fabricated by laser scanning. The result indicated that bubble prints of liquid colloids were demonstrated for the first time, as far as we know. Furthermore, the conductivity was evaluated using a four-terminal method with conductive adhesive on a gold wire in the squares at both ends of the dumbbell pattern, and the result was that high resistance (>1 GΩ) and conductivity could not be obtained. The conductivity is low compared to the bulk conductivity of EGaIn (3.4 × 10^6^ S/m [28]), which suggests that the conductivity of the fabricated wiring itself is lowered or that the wiring is defective. As no obvious defects were noticed when the wiring was observed under high magnification with SEM (Figure 4c), the resistivity of the wiring itself formed with liquid metal colloids was considered high.

### 3.3. Improving the Electrical Conductivity of EGaIn Colloid Wires Using Galvanic Displacement

**Figure 5:**
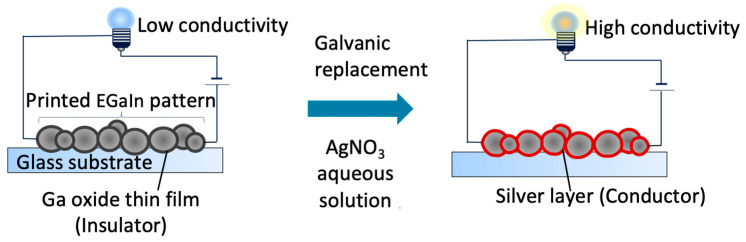
Illustration of the improved conductivity of a fabricated line using galvanic displacement.

Previous studies have reported that EGaIn colloidal particles have a 0.5–1 nm thick layer of gallium oxide (α-Ga_2_O_3_ or β-Ga_2_O_3_) on their surface [43]. Although EGaIn is conductive, gallium oxide is an ultra-wide-bandgap semiconducting material [45]. Therefore, the conductivity of the fabricated pattern should be considered low [46]. David et al. used galvanic replacement, a substitution method that utilizes the difference in the reduction potentials of metals [47] to replace the gallium oxide layer on the surface of EGaIn with a conductive metal, to fabricate highly conductive particles [43]. However, this method cannot be directly adapted for bubble printing. Their method used water as a solvent during the galvanic replacement of particles; however, as mentioned above, water-based bubble printing is difficult to pattern in this system. Galvanic replacement was not confirmed when NMP was used as the solvent (Figure S3). Unlike David et al. [43], we considered a method of galvanic replacement after pattern formation (Figure 5) instead of direct galvanic replacement on the particles. This is because NMP is better suited for bubble printing in a solvent, whereas galvanic replacement is better suited for an aqueous solution.

**Figure 6:**
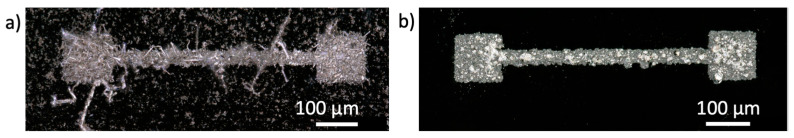
Photographs of the fabricated line after galvanic replacement at different concentrations of AgNO_3_: (**a**) 0.5 M and (**b**) 12.7 M. Immersion time: 24 h.

Galvanic replacement was performed by immersing the dumbbell pattern in aqueous AgNO_3_ solution for 24 h. Because the reduction potential of gallium (E° = −0.549 V [48]) is lower than that of silver (E° = 0.7996 V [48]) in galvanic replacement, gallium is oxidized and converted to gallium ions, while silver ions are reduced and deposited on the particle surface [47,49]. Figure 6 shows the images of the patterns when immersed in aqueous AgNO_3_ solutions of different concentrations. The sample immersed in 0.5 M AgNO_3_ formed numerous needle-like structures on the pattern, while the sample immersed in 12.7 M AgNO_3_ solution maintained the shape of the pattern. The value of 12.7 M was chosen to be close to a saturated aqueous solution of silver nitrate at 20 °C. EDX spectra confirmed that, during galvanic replacement, the formed pattern in gallium oxide of the EGaIn colloid surface was replaced with silver (Figures S4 and S5). These results are consistent with the report of Hoshyargar et al. that the surface morphology of EGaIn after galvanic replacement varies with the concentration of the AgNO_3_ solution [49]. David et al. explained that the surface structure changes with concentration because galvanic replacement involves two processes: nucleation and polycrystal growth [43]. Galvanic replacement at high concentrations favors the fabrication of wiring. This is because, during galvanic replacement at a low concentration, the needle-like structures grow outside the fabricated pattern, reducing the accuracy of modeling and increasing the likelihood of short circuits. The conductivities of the samples galvanically substituted with 0.5 M AgNO_3_ and 12.7 M AgNO_3_ were (6.1 ± 1.6) × 10^4^ and (1.4 ± 0.6) × 10^6^ S/m, respectively. Conductivity is greatly enhanced after galvanic replacement, possibly because of the replacement of gallium oxide with silver, which acts as a conductor. The growth of silver polycrystals in galvanic substitution may have improved conductivity due to reduced internal defects.

**Figure 7:**
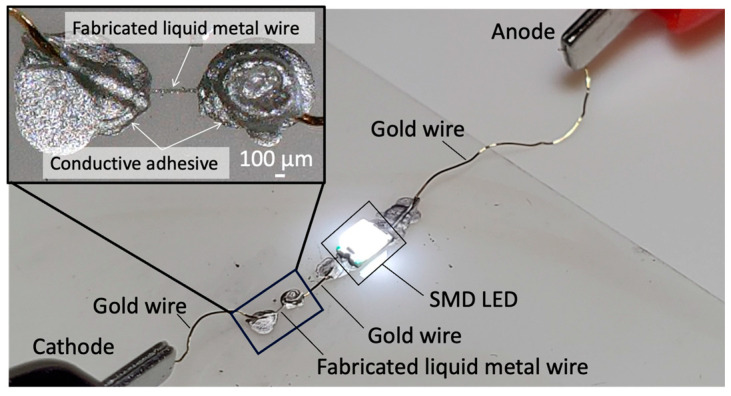
Confirmation that the fabricated line functions as wiring from the lighting of the SMD LED. Immersed in 12.7 M AgNO_3_ for 24 h.

To demonstrate that liquid metal colloid wiring is conductive, electronic circuits to light up SMD LEDs were formed using conductive EGaIn patterns obtained by galvanic replacement with 12.7 M AgNO_3_. This confirmed that the EGaIn patterns can function as wiring, as shown in Figure 7 and Figure S6.

The EDX peak for Ga disappears at 12.7 M. In the case of immersion in 12.7 M AgNO_3_, no gallium-derived peaks were detected, even when the EDX acceleration voltage was increased to 20 kV (Figure S7). The X-ray production range of silver with an acceleration voltage of 20 kV from Castaing’s equation [50] is 1.1 µm. The X-ray production range was larger when calculated using Ga or In. All the gallium is estimated to be replaced by silver because the fabrication line is approximately 1 µm thick (Figure S2). In the EDX spectrum of the fabricated line, a silicon peak originating from the glass substrate was observed at an acceleration voltage of 20 kV (Figure S7), indicating that the thickness of the fabricated line was analyzed. In addition, indium peaks were observed, which remained within the fabricated line. The conductivity of the sample immersed in 12.7 M is lower than that of silver (6.30 × 10^7^ S/m [51]). As the wiring is formed by agglomerating particles that are not in complete contact with each other, areas where they are in contact may exist, and this may reduce the conductivity. Although conductive wiring has been successfully fabricated, we expected that wiring fabricated under these conditions would have difficulty combining with the flexibility derived from liquid metal.

Next, we examined the conditions under which conductivity and flexibility could be expected. The immersion time in 12.7 M AgNO_3_ varied from 24 h to 1, 3, 5, and 10 min. After the immersion for 1, 3, and 5 min, both Ag and Ga peaks were observed in the EDX spectrum (Figures S8–S10), and it was assumed that the wiring structure was formed with only the surface replaced by silver. However, when the immersion time was increased to 10 min, EDX mapping showed that gallium was lost at many locations, although the remaining areas of gallium could be detected (Figure S11). Longer immersion times were associated with a lower Ga content. The conductivity of each sample was then evaluated. Two of the six samples immersed for 1 min in AgNO_3_ were not conductive, and even those that were conductive had a maximum conductivity of 1.2 × 10^3^ S/m. This is considered to be owing to the insufficient galvanic displacement and low conductivity at an immersion time of 1 min. After 3, and 5, and 10 min of immersion, the conductivities were (7.1 ± 11) × 10^4^, (1.5 ± 2.0) × 10^5^, and (7.9 ± 5.4) × 10^5^ S/m, respectively. Higher conductivity was obtained with longer silver nitrate immersion times. This is consistent with the increase in the intensity of silver in EDX. The EDX results and conductivity measurements indicated that the sample with 5 min galvanic replacement at 12.7 M was the best sample in achieving both flexibility and conductivity. This is because sufficient EGaIn remained to provide flexibility and conductivity. It is considered sufficiently conductive for wiring use. Under these conditions, we believe that EGaIn colloidal particles with conductive metal shells, similar to those reported by David et al. [43], form a line-like structure, as shown in Figure 5.

**Figure 8:**
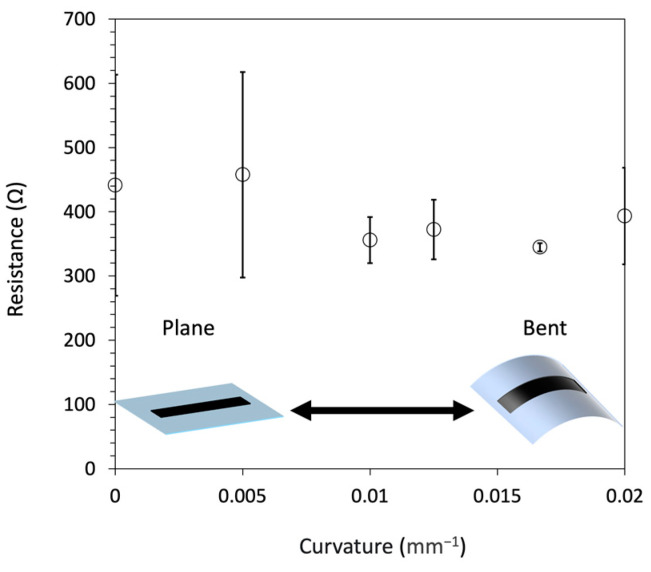
Dependence of the resistance on the curvature of the flexible wiring fabricated by the bubble printing of EGaIn colloidal particles and galvanic replacement. Immersed in 12.7 M AgNO_3_ for 5 min.

To demonstrate flexible wiring, we used an ultra-thin glass substrate (G-Leaf OA-10G, Nippon Electric Glass Co., Ltd., Otsu, Siga, Japan) with a thickness of 100 µm, which has excellent properties such as high flexibility, high hardness, and optical transparency. Specifically, EGaIn colloidal particles were bubble printed to fabricate wiring, immersed in 12.7 M AgNO_3_ for 5 min, and then deformed at curvatures of 0, 0.050, 0.010, 0.013, 0.017, and 0.020 mm^−1^ to measure the resistance. A jig with the specified curvature was fabricated using a fused deposition modeling (FDM)-type 3D printer (Guider 2, Flashforge Technology Co., Ltd., Jinhua, Zhejiang, China) and bent by fixing flexible glass with fabricated wiring. The results showed no significant change in the resistance to wire bending (Figure 8). It was confirmed that the conductivity was maintained against bending, and flexible wiring was achieved. In addition, the flexible glass itself was destroyed at a curvature of 0.025 mm^−1^, so the wiring itself could withstand bending of more than 0.020 mm^−1^ in curvature.

### 3.4. Fabrication of a Fine Line Pattern

**Figure 9:**
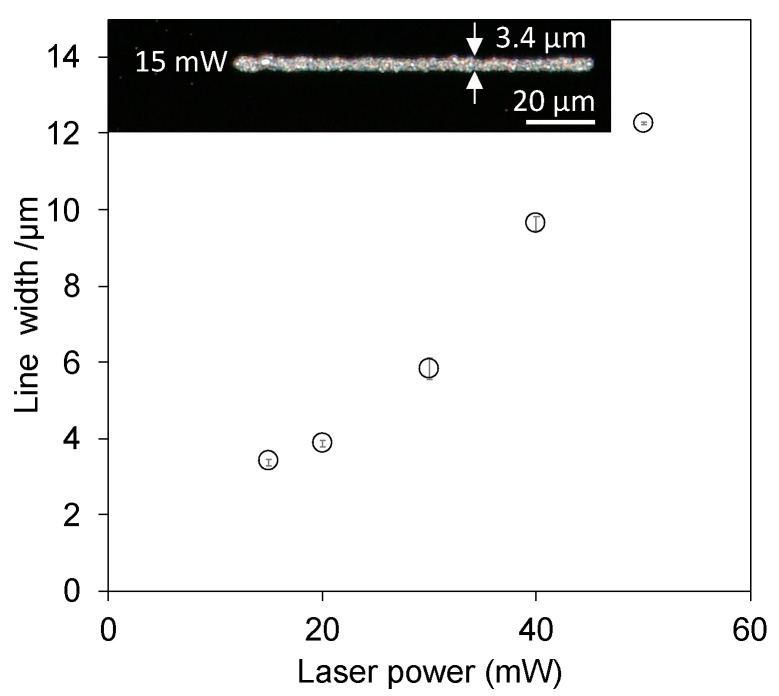
Relationship between the laser power and fabricating line width. The inset shows lines fabricated at 15 mW.

Because controlling the laser power is considered to change the bubble diameter and determine the line width of the pattern, we investigated the minimum laser power required to generate bubbles and accumulate the particles on the substrate. Figure 9 shows the linewidths of the patterns at different laser powers ranging from 15 to 50 mW. The minimum line width of 3.4 µm was achieved at 15 mW, which was the lowest laser power that enabled bubble printing. The line width could be controlled by increasing the laser power.

## 4. Conclusions

NMP dispersions of EGaIn colloidal particles were obtained by ultrasonic irradiation. The diameter of the particles was confirmed to be approximately 1 µm using DLS and SEM. Direct laser wiring using a femtosecond laser was performed with the NMP dispersion of EGaIn colloidal particles. Bubble prints of liquid colloids were demonstrated for the first time, as far as we know. SEM-EDX and CCD observations confirmed that the microbubbles generated by irradiating the NMP dispersion of the EGaIn colloidal particles by a femtosecond laser beam caused the deposition of particles on the glass substrate according to the trajectory of the laser scanning. Because the gallium oxide layer on the surface of the EGaIn colloidal particles functions as a resistance layer, we replaced gallium oxide with silver through galvanic replacements. As a result, optimized conductive wiring with a conductivity of (1.5 ± 2.0) × 10^5^ S/m was successfully fabricated. In addition, the ability to achieve both conductivity and flexibility was demonstrated by a bending test with wiring fabricated on a flexible glass substrate. Since the flexibility of glass substrates is limited, more flexible organic films will be considered in the future. Furthermore, the optimization of the laser power in bubble printing enabled the wiring of a fine line pattern with a minimum line width of 3.4 µm. The proposed method can be used to fabricate flexible conductive wiring consisting of liquid metal colloids, thereby allowing the fabrication of sophisticated flexible electronic devices such as sensors and batteries.

## Data Availability

The original contributions presented in the study are included in the article and Supplementary Materials; further inquiries can be directed to the corresponding author.

## Supplementary Materials

The following supporting information can be downloaded at https://www.mdpi.com/article/10.3390/nano14201665/s1. Table S1: Comparison of this study with previous papers on bubble printing with conductive materials; Figure S1: Scanning electron microscope (SEM) image and dynamic light scattering (DLS) histogram of gallium–indium eutectic alloy (Ga-In) particles; Figure S2: Example of height evaluation of the fabricated line using a laser scanning microscope; Figure S3: Energy-Dispersive X-ray Spectroscopy (EDX)-SEM image of a sample in which silver nitrate (AgNO_3_) was added to NMP to attempt galvanic replacement of the surface of Ga-In colloidal particles. EDX mapping of Silver (Ag) on Ga-In colloidal particles has not been observed, and experiments using Ga-In colloidal particles in *N*-methylpyrrolidone (NMP) confirm the difficulty of galvanic replacement of the colloidal particles. Acceleration voltage: 6 kV; Figure S4: EDX spectra of the inside and outside of the processed line for galvanic replacement with 0.5 M AgNO_3_ for 24 h and the corresponding EDX mapping of gallium (Ga) and Ag. Acceleration voltage: 6 kV; Figure S5: EDX spectra of the inside and outside of the processed line for galvanic replacement with 12.7 M AgNO_3_ for 24 h and the corresponding EDX mapping of Ga and Ag. Acceleration voltage: 6 kV; Figure S6: Off state of the fabricated wiring and SMD LED; Figure S7: EDX spectra of the inside and outside of the processed line for galvanic replacement with 12.7 M AgNO_3_ for 24 h and the corresponding EDX mapping of Ga and Ag. Acceleration voltage: 20 kV; Figure S8: EDX spectra of the inside and outside of the processed line for galvanic replacement with 12.7 M AgNO_3_ for 1 min and the corresponding EDX mapping of Ga and Ag. Acceleration voltage: 6 kV; Figure S9: EDX spectra of the inside and outside of the processed line with galvanic replacement with 12.7 M AgNO_3_ for 3 min and the corresponding EDX mapping of Ga and Ag. Acceleration voltage: 6 kV; Figure S10: EDX spectra of the inside and outside of the processed line with galvanic replacement with 12.7 M AgNO_3_ for 5 min and the corresponding EDX mapping of Ga and Ag. Acceleration voltage: 6 kV; Figure S11: EDX spectra of the inside1, inside 2, and outside of the processed line with galvanic replacement with 12.7 AgNO_3_ for 10 min and the corresponding EDX mapping of Ga and Ag. Acceleration voltage: 6 kV; Supplemental Video S1: Microbubble generation was observed in the EGaIn colloidal solution dispersed in NMP and agglomerated particles were observed after laser scanning; Supplemental Video S2: Laser irradiation of NMP solution; Supplemental Video S3: Excess microbubble generation in liquid metal colloidal solution dispersed in water.
