# Supplementary material for: Bubble Printing of Liquid Metal Colloidal Particles for Conductive Patterns
Source: Nanomaterials (Basel). 2024 Oct 17;14(20):1665. doi: 10.3390/nano14201665 (PMC11510549; doi:10.3390/nano14201665)
Supplement: Supplementary file 1 [file nanomaterials-14-01665-s001.zip › SI_proof_check_final.pdf]

Bubble printing of liquid metal colloidal particles for conductive patterns

Masaru Mukai[1], Tatsuya Kobayashi[2], Mitsuki Sato[2], Juri Asada[2], Kazuhide Ueno[1], Taichi Furukawa[1] and Shoji Maruo[1]*

1. Faculty of Engineering, Yokohama National University, 79-5 Tokiwadai, Hodogaya-ku, Yokohama 240-8501, Japan
2. Graduate School of Engineering Science, Yokohama National University, 79-5 Tokiwadai, Hodogaya-ku, Yokohama 240-8501, Japan

(*Electronic mail: maruo-shoji-rk@ynu.ac.jp)

Table S1. Comparison of this study to previous studies on bubble printing using conductive materials.

	Edri (2021) et al.	Edri (2020) et al.	Nishiyama (2019) et al	Roy (2013) et al.	Armon (2021) et al.	This work
Representative conductivity	3.1×10^5 S/m (Calculated)	3.8 x 10 S/m (Calculated, HCl dope)	Not described	Not described	1.0×10^6 S/m (Calculated, Sintering with HCl)	1.5×10^5 S/m
Scan speed	100 μm/s	200 ～ 800 μm/s	40 ～ 900 μm/s	>1 mm/s	10 μm/s ～ 9 mm/s	800 μm/s
Maximum Resolution （width)	≅ 4 μm	650 nm	2.0 μm	≅ 4 μm	1.7 μm	3.4 μm
Method	Bubble printing + thermochemical reaction	Bubble printing	Bullle printing + Photochemical reaction	Bubble printing	Bubble printing	Bubble printing
Dispersant	$PtCl_2$	Conductive polymer (polyaniline)	$AgNO_3$ + (TiO_2,SiO_2, Fe_2O_3)	Carbon nanotube (CNT) +Soft oxometalate (SOM)	Ag particles	Ga-In (Galvanic replacement)
Laser souce	532 nm (CW)	532 nm (CW)	780 nm (Femtosecond laser)	1064 nm (CW)	532 nm (CW)	752 nm (Femtosecond laser)
Dispersion medium	NMP	NMP	EtOH + H_2O	H_2O	Diethylene glycol butyl ether	NMP
Reference No. (DOI:)	[27] (https://doi.org/10.1021/acsami.1c06204)	[24] (https://doi.org/10.1021/acsami.0c00904)	[26] (https://doi.org/10.1038/s41598-019-50630-1)	[25] (https://doi.org/10.1021/la402777e)	[13] (https://doi.org/10.1021/acsami.7b14614)	-

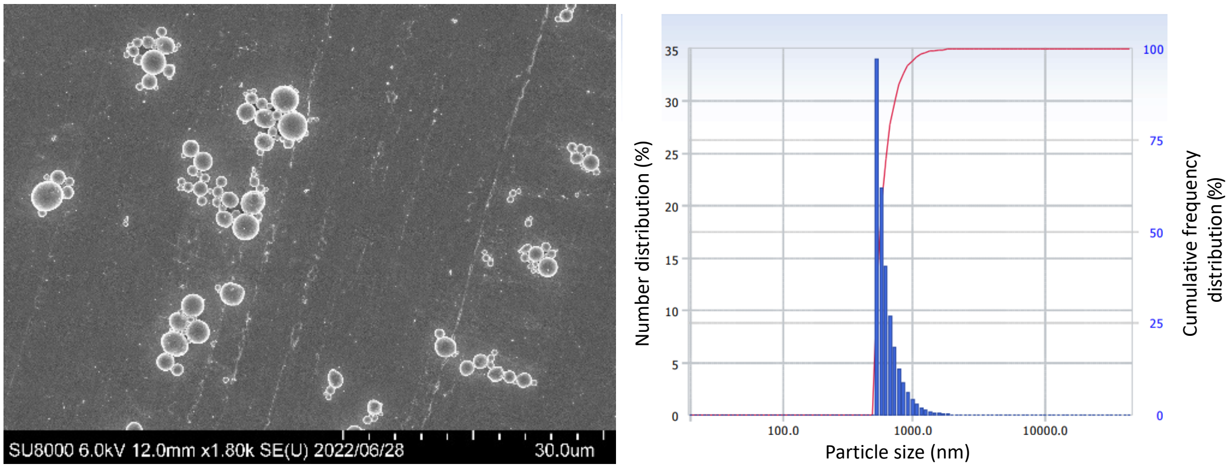

Figure S1. Scanning electron microscope (SEM) image and dynamic light scattering (DLS) histogram of gallium-indium eutectic alloys (Ga-In) particles.

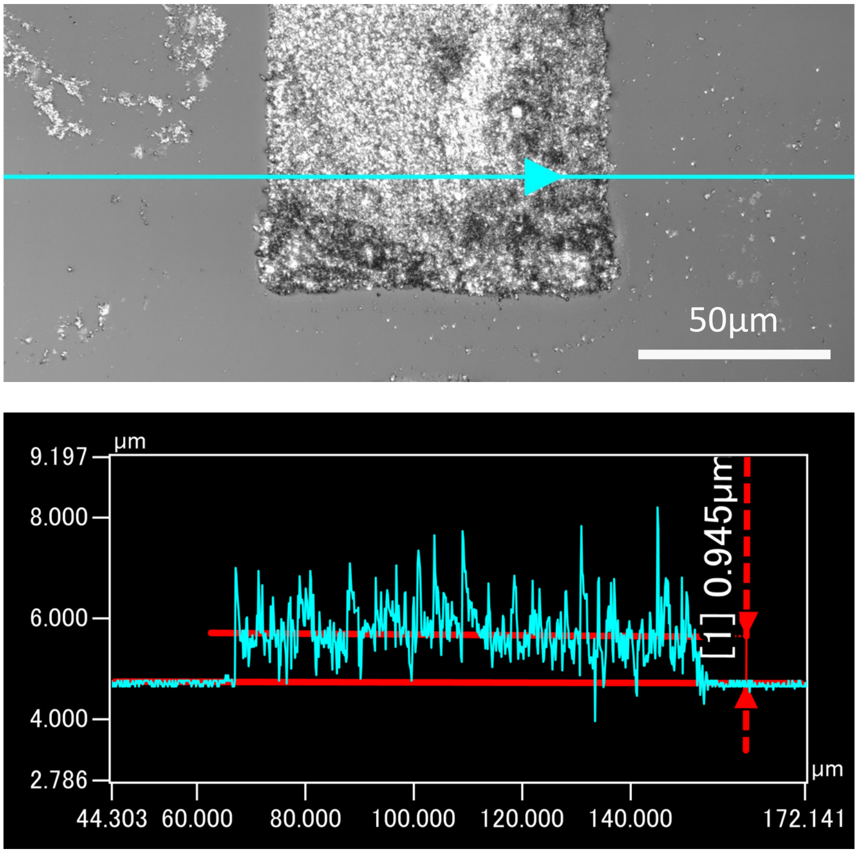

Figure S2. Example of height evaluation of the fabricated line using a laser scanning microscope.

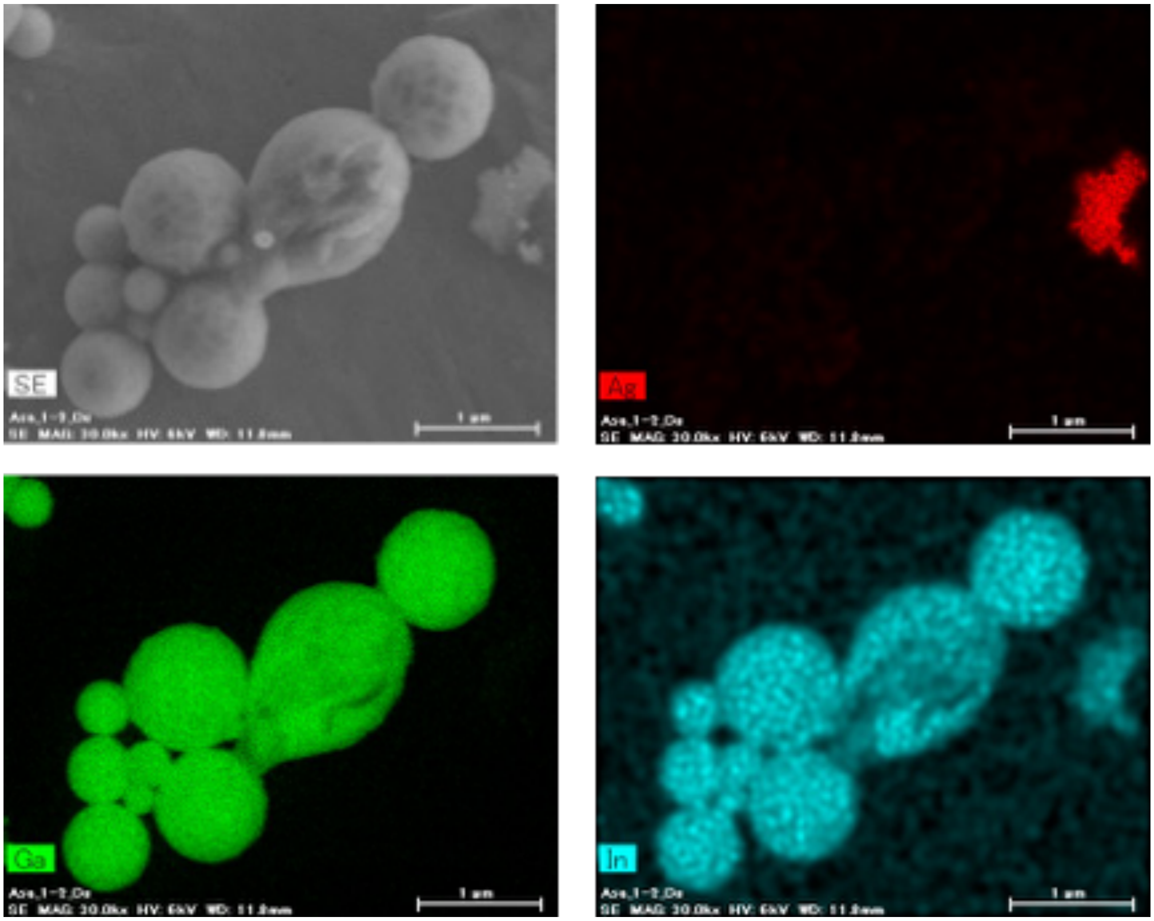

Figure S3. Energy Dispersive X-ray Spectroscopy (EDX) -SEM image of a sample in which silver nitrate ($AgNO_3$) was added in NMP to attempt galvanic replacement of the surface of Ga-In colloidal particles. EDX mapping of Silver (Ag) on Ga-In colloidal particles has not been observed, and experiments using Ga-In colloidal particles in *N*-methylpyrrolidone (NMP) confirm the difficulty of galvanic replacement of the colloidal particles. Acceleration voltage: 6 kV.

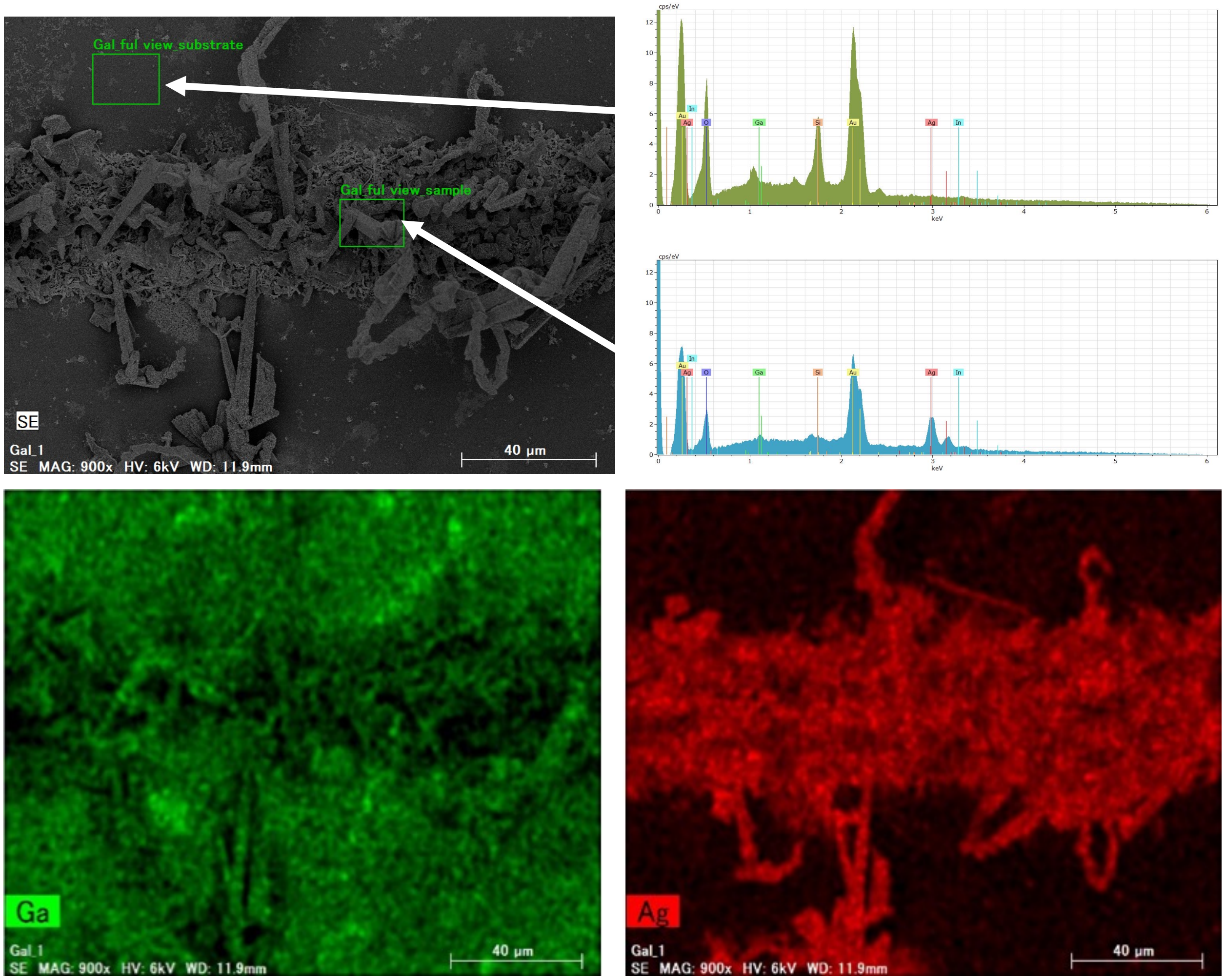

Figure S4. EDX spectra of the inside and outside of the processed line for galvanic replacement with 0.5 M $AgNO_3$ for 24 h and the corresponding EDX mapping of gallium (Ga), and Ag. Acceleration voltage: 6 kV.

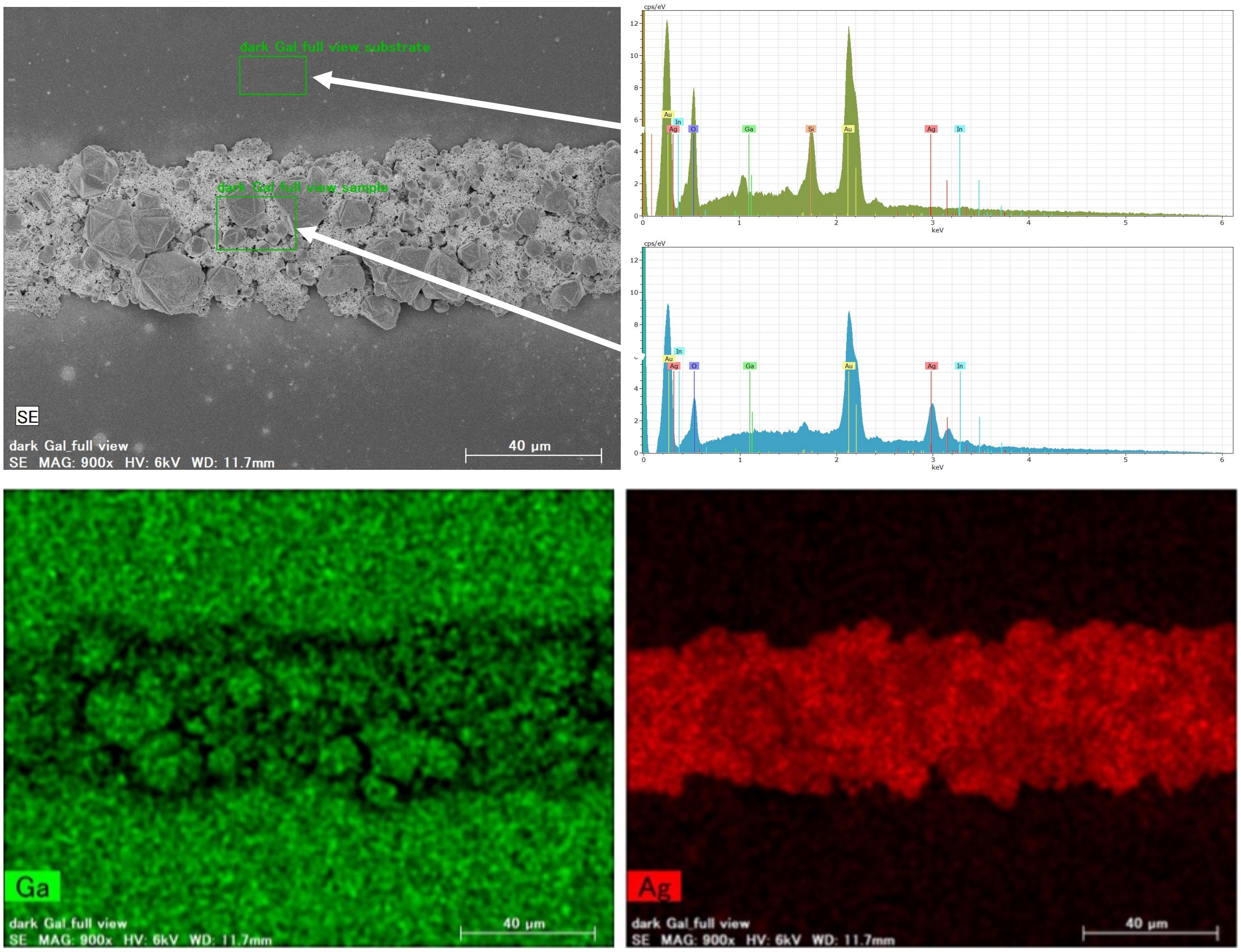

Figure S5. EDX spectra of the inside and outside of the processed line for galvanic replacement with 12.7 M $AgNO_3$ for 24 h and the corresponding EDX mapping of Ga, and Ag. Acceleration voltage: 6 kV.

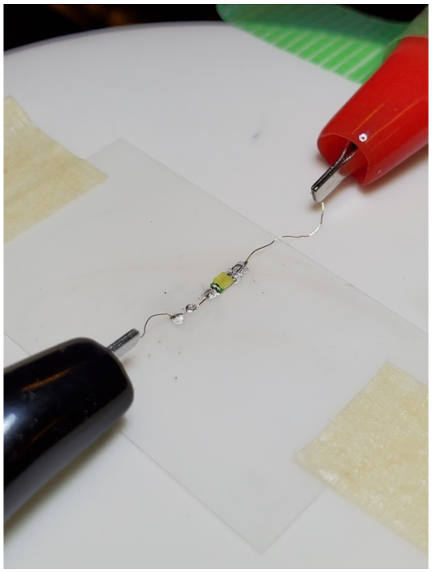

Figure S6. Off state of the fabricated wiring and SMD LED.

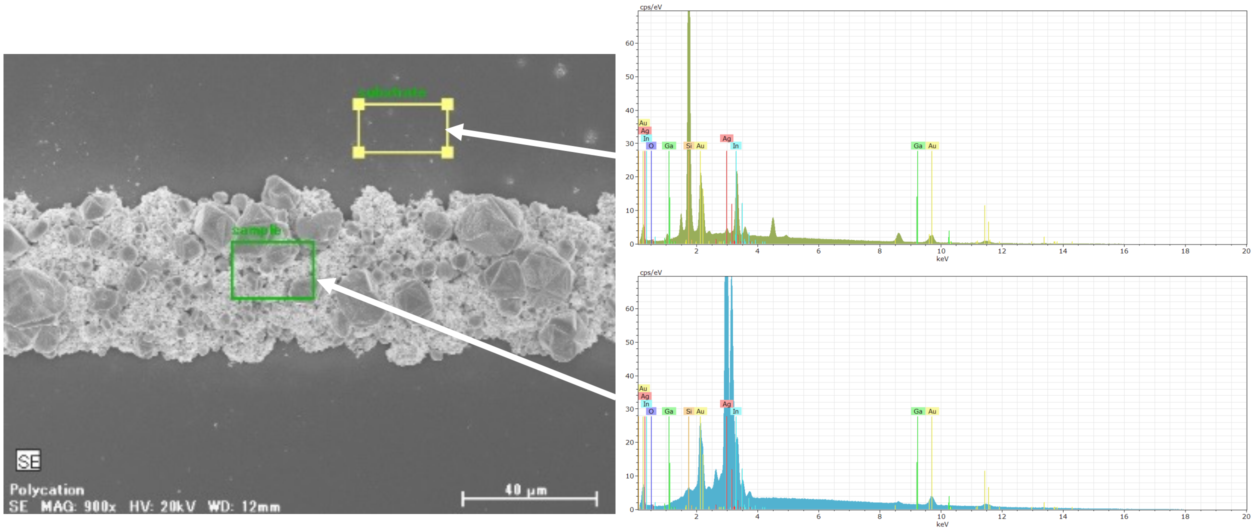

Figure S7. EDX spectra of the inside and outside of the processed line for galvanic replacement with 12.7 M $AgNO_3$ for 24 h and the corresponding EDX mapping of Ga, and Ag. Acceleration voltage: 20 kV.

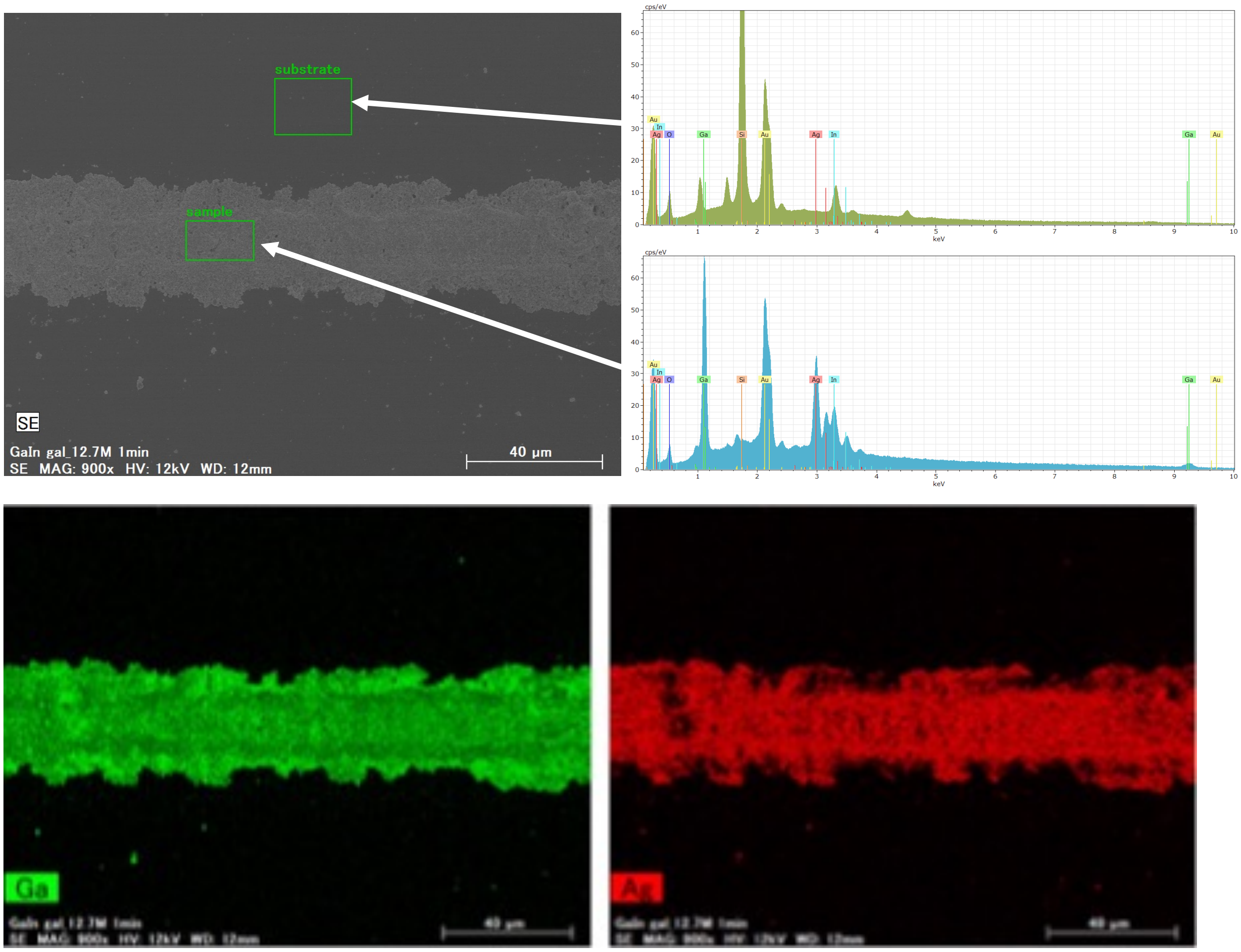

Figure S8. EDX spectra of the inside and outside of the processed line for galvanic replacement with 12.7 M $AgNO_3$ for 1 min and the corresponding EDX mapping of Ga, and Ag. Acceleration voltage: 6 kV.

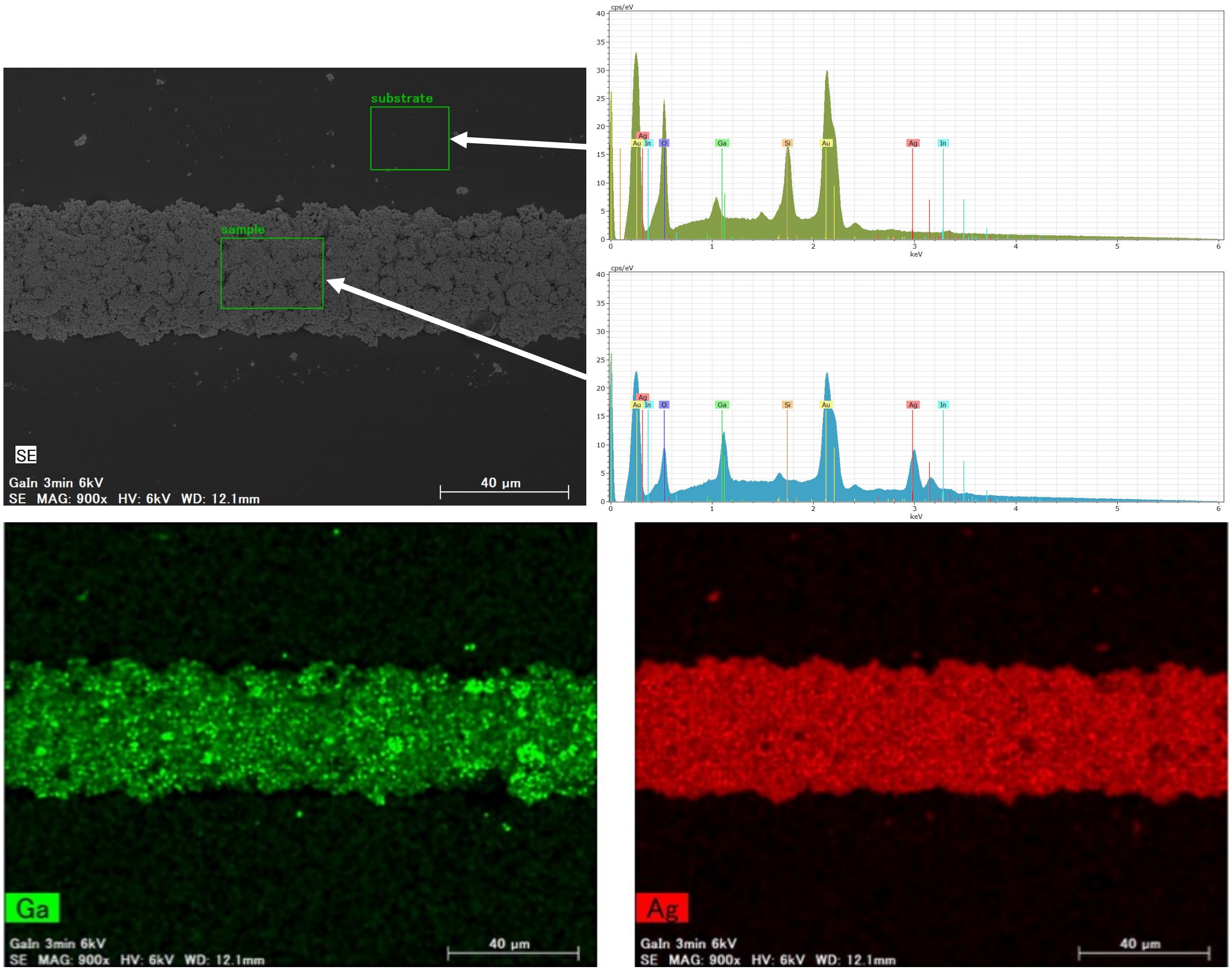

Figure S9. EDX spectra of the inside and outside of the processed line with galvanic replacement with 12.7 M $AgNO_3$ for 3 min and the corresponding EDX mapping of Ga and Ag. Acceleration voltage: 6 kV.

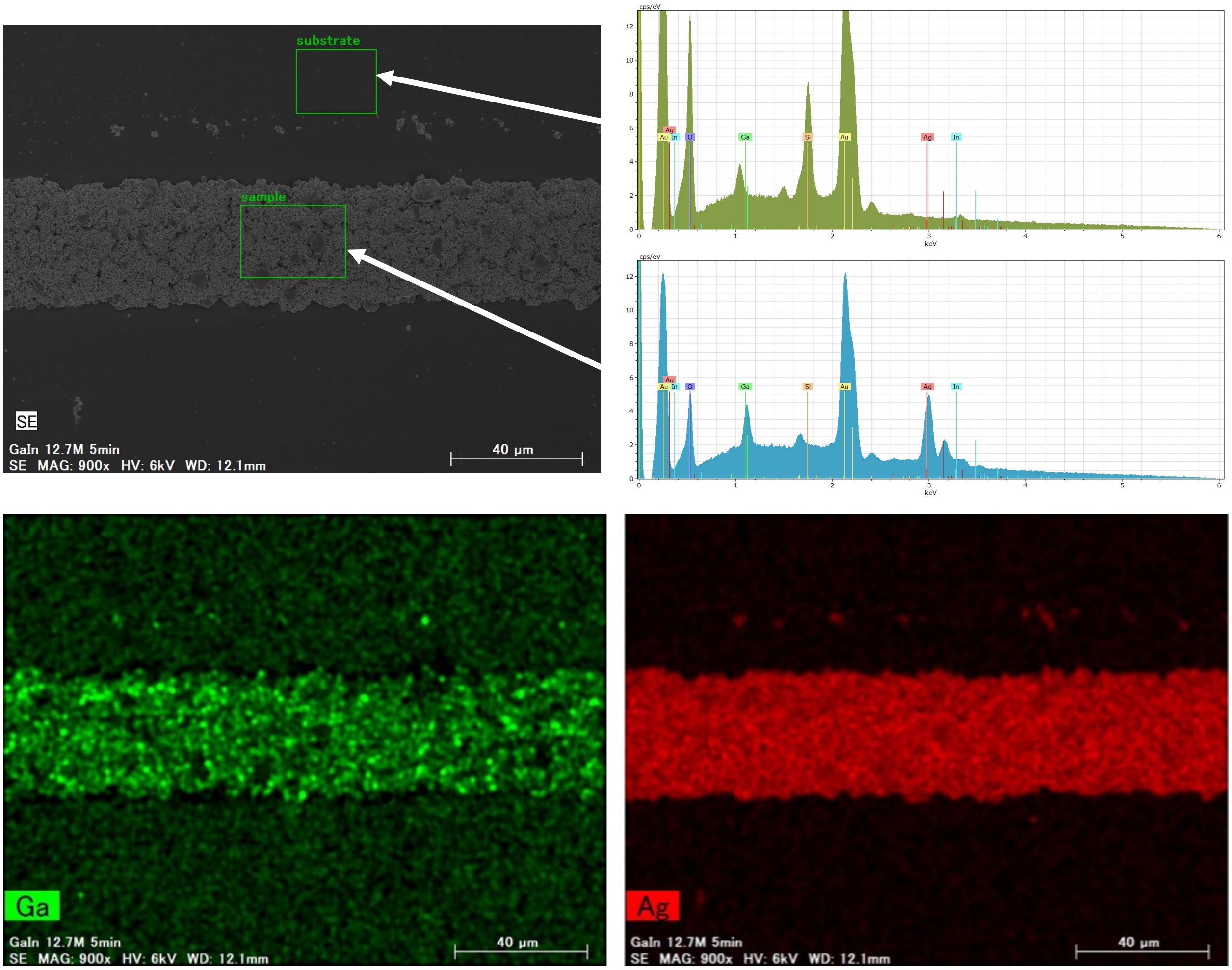

Figure S10. EDX spectra of the inside and outside of the processed line with galvanic replacement with 12.7 M $AgNO_3$ for 5 min and the corresponding EDX mapping of Ga, and Ag. Acceleration voltage: 6 kV.

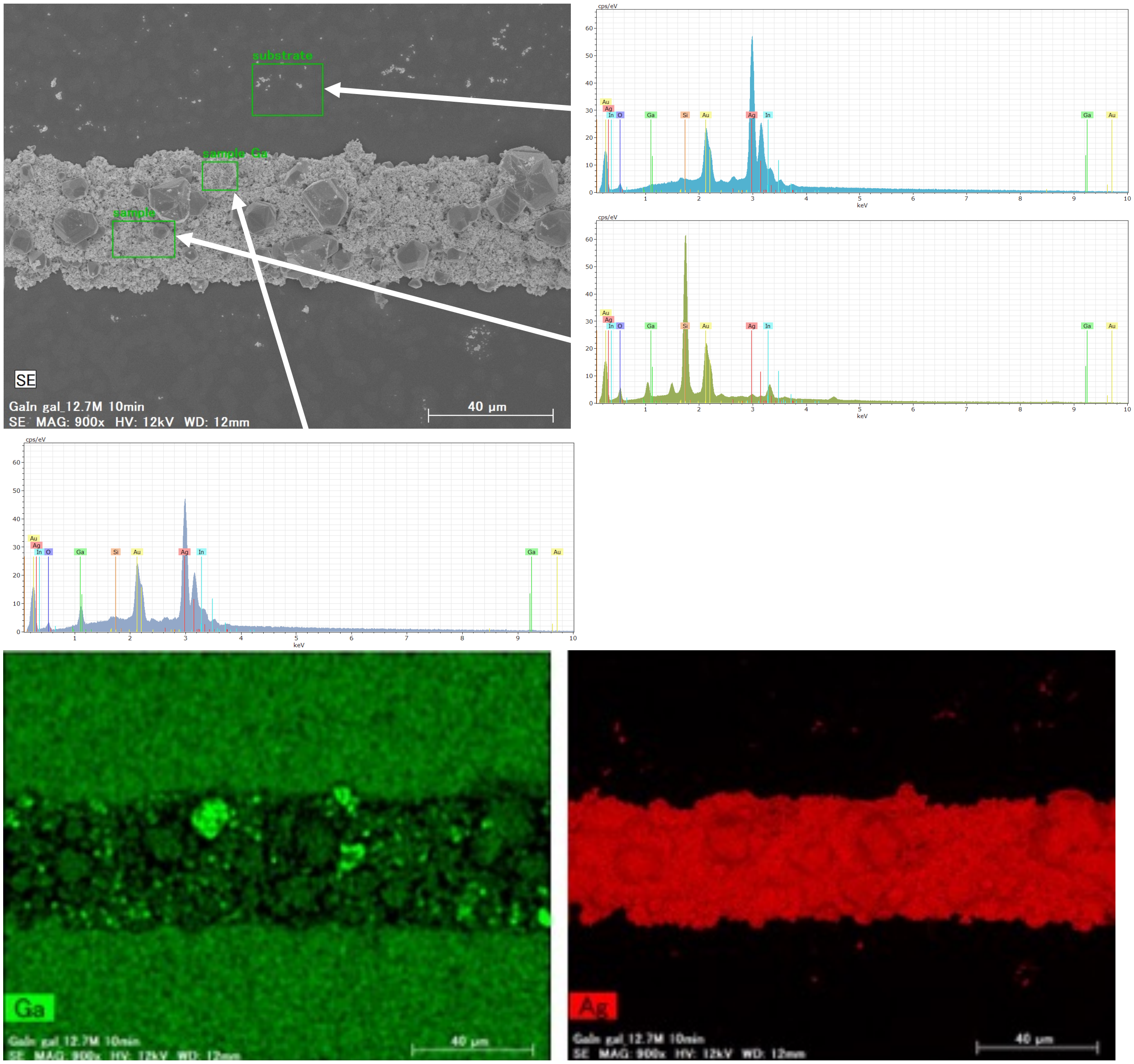

Figure S11. EDX spectra of the inside1, inside 2, and outside of the processed line with galvanic replacement with 12.7 $AgNO_3$ for 10 min and the corresponding EDX mapping of Ga and Ag. Acceleration voltage: 6 kV.
